# Divergence in Coding Sequence and Expression of Different Functional Categories of Immune Genes between Two Wild Rodent Species

**DOI:** 10.1093/gbe/evab023

**Published:** 2021-02-10

**Authors:** Xiuqin Zhong, Max Lundberg, Lars Råberg

**Affiliations:** Department of Biology, Lund University, Sweden

**Keywords:** *Apodemus flavicollis*, gene expression evolution, *Myodes glareolus*, protein evolution, wild immunology

## Abstract

Differences in immune function between species could be a result of interspecific divergence in coding sequence and/or expression of immune genes. Here, we investigate how the degree of divergence in coding sequence and expression differs between functional categories of immune genes, and if differences between categories occur independently of other factors (expression level, pleiotropy). To this end, we compared spleen transcriptomes of wild-caught yellow-necked mice and bank voles. Immune genes expressed in the spleen were divided into four categories depending on the function of the encoded protein: pattern recognition receptors (PRR); signal transduction proteins; transcription factors; and cyto- and chemokines and their receptors. Genes encoding PRR and cyto-/chemokines had higher sequence divergence than genes encoding signal transduction proteins and transcription factors, even when controlling for potentially confounding factors. Genes encoding PRR also had higher expression divergence than genes encoding signal transduction proteins and transcription factors. There was a positive correlation between expression divergence and coding sequence divergence, in particular for PRR genes. We propose that this is a result of that divergence in PRR coding sequence leads to divergence in PRR expression through positive feedback of PRR ligand binding on PRR expression. When controlling for sequence divergence, expression divergence of PRR genes did not differ from other categories. Taken together, the results indicate that coding sequence divergence of PRR genes is a major cause of differences in immune function between species.

**Significance**

Immunity to pathogens often differs considerably between host species, but the genetic basis of such differences is not well understood. To address this issue, we here compared protein-coding sequences and expression of different types of immune genes between two rodent species. Our results indicate that divergence of protein-coding sequences of one particular type of immune genes—receptors involved in the initial recognition of pathogens—play a key role in differences in immune function between host species.

## Introduction

There are often substantial differences between host species in disease severity when infected by a given pathogen. One example that has gained considerable interest concerns Ebola virus, where bats seem to carry the virus more or less asymptomatically (Leroy et al. 2005), while infection causes severe disease in humans. Similarly, but on a smaller phylogenetic scale, different primate species differ in susceptibility to simian immunodeficiency virus infection. Macaques develop AIDS-like symptoms, whereas sooty mangabeys do not (Mandl et al. 2015). Such differences in susceptibility to infectious diseases could be a result of numerous factors, but differences in immune function likely plays the most important role (Mandl et al. 2015). To understand how differences in immune function between species have evolved, it would be of interest to uncover general principles of divergence of immune signaling pathways, for example if divergence in protein coding sequence and/or gene expression is concentrated to certain functional categories of immune genes.

Analyses of coding sequences in invertebrates (*Drosophila*) have shown that some types of immune genes, in particular receptors involved in detection of pathogens, are more often under positive selection than other genes in immune pathways (encoding signal transduction and effector proteins) (Sackton et al. 2007), and thus could be expected to have higher coding sequence divergence. Similar patterns have been found in primates, where proteins at the margins of immune networks (e.g.,pattern recognition receptors [PRR], which are involved in recognition of pathogens) have higher divergence than core proteins (e.g., signal transduction proteins [Casals et al. 2011]).

A recent analysis of in vitro expression of mammalian (rodent and primate) immune genes in response to stimulation with viral or bacterial ligands showed that also interspecific divergence in expression was concentrated to a few functional categories of genes (Hagai et al. 2018). Specifically, expression of PRR as well as cyto- and chemokines and their receptors (which are involved in intercellular communication) had high divergence. In contrast, there was low divergence in expression of genes encoding intracellular signal transduction proteins (e.g., protein kinases, adaptors, inhibitors, etc.) and transcription factors (Hagai et al. 2018).

Thus, analyses of interspecific divergence in coding sequence or expression in different study systems have shown that in both cases some functional categories of immune genes have higher divergence than others. However, previous studies have focused on either coding sequence or expression divergence, used different study systems, and different ways of categorizing immune genes. It is therefore not entirely clear whether differences in coding sequence and expression divergence between gene categories are concordant or not. To understand how differences in immune function between species have evolved, it would be of interest to compare both coding sequence and expression divergence of different immune gene categories in a given system and elucidate how these two aspects of divergence are related to each other. For example, do some gene categories have high divergence as regards both coding sequence and expression, or do some categories have high sequence divergence while others have high expression divergence?

Moreover, it is not well known if differences in divergence between functional categories occur independently of other factors or not. Under one scenario, differences in coding sequence or expression divergence could be an effect of that natural selection has acted in different ways on different gene categories because of their function per se*.* For example, one could envision that receptors for recognition of pathogens might have high sequence divergence because these receptors are involved in recognition of specific pathogens and each host species has adapted to its own sets of pathogens (Sackton et al. 2007; Lundberg et al. 2020). Alternatively, gene categories might differ in sequence or expression divergence because they differ in other factors that covary with sequence or expression divergence, so that once these other factors are controlled for there is no difference in divergence between gene categories. For example, sequence divergence is generally negatively correlated with pleiotropy (Zhang and Yang 2015), and receptors for recognition of pathogens have relatively low pleiotropy (Lundberg et al. 2020). Thus, receptors could have high sequence divergence because they have low pleiotropy, rather than because they are involved in recognition of pathogens (Sackton et al. 2007)*.* Besides pleiotropy, coding sequence divergence is correlated with several other factors, in particular expression level (Zhang and Yang 2015). Similarly, expression divergence has been found to be associated with both pleiotropy and expression level (Hagai et al. 2018, Warnefors and Kaessmann 2013). These factors could thus potentially confound any differences in sequence or expression divergence between gene categories. However, as far as we are aware, the relative importance of gene function and other factors for coding sequence or expression divergence of immune genes has not been explicitly addressed.

Here, we use two wild rodent species—the bank vole and the yellow necked mouse—to investigate both coding sequence and expression divergence of genes in immune signaling pathways. Specifically, we tested if there are differences in coding sequence divergence and expression divergence between functional categories of immune genes, and if differences in divergence between categories occur independently of other factors (pleiotropy and expression level) or not. To this end, we generated de novo transcriptome assemblies and expression data from spleen of wild-caught bank voles and yellow-necked mice.

## Methods and Materials

### Study Species and Field Work

The bank vole (*Myodes glareolus*, Schreber 1780) is a small rodent (adult body mass 15–40 g) in the family Cricetidae (hamsters, lemmings, voles, etc.), which occurs in forests and meadows with thick ground cover, from western Europe to central Siberia (Aulagnier et al. 2009; Wilson et al. 2017). The yellow-necked mouse (*Apodemus flavicollis*,Melchior 1834) is a small rodent (adult body mass 22–56 g) in the family Muridae (rats and mice), which occurs in forests in temperate parts of Europe (Aulagnier et al. 2009; Wilson et al. 2017). Cricetidae and Muridae diverged ca. 18 Ma (Steppan & Schenk 2017).

The bank vole and yellow-necked mouse share pathogens to a large extent. At our study site, both species are infested with ticks (*Ixodes ricinus*), and infected with the tick-transmitted bacteria *Borrelia afzelii* and *Candidatus* Neoehrlichia mikurensis (Andersson and Råberg 2011; Hellgren et al. 2011) and various helminths (Clough and Råberg 2014). The bank vole and the yellow-necked mouse differ in resistance to at least some of these pathogens. Bank voles have about 10-fold higher infection intensities with *B. afzelii* (i.e., bacterial load in infected animals) than yellow-necked mice (Råberg 2012; Strandh and Råberg 2015), even though the two species carry the same *B. afzelii* strains (Råberg et al. 2017). In contrast, yellow-necked mice have considerably higher tick burdens than bank voles living in the same area (L. Råberg, unpublished data), which is likely an effect of that voles but not mice develop acquired resistance to ticks (Dizij and Kurtenbach 1995).

In previous analyses of gene expression in spleen of wild-caught naturally *B. afzelii*-infected and uninfected bank voles and yellow-necked mice, we found that the two species respond in partly different ways to *B. afzelii* infection. Specifically, *B. afzelii* infection is associated with up-regulation of IFNα-signaling in yellow-necked mice, but down-regulation of IL6 signaling and the complement system pathway in bank voles (Zhong et al. 2020). These differences in immune response presumably contribute to the observed difference in *B. afzelii* infection intensity between the two species (Råberg 2012; Strandh and Råberg 2015). In the present study, we use the dataset from Zhong et al. (2020), but instead of focusing on differences between the two species in the response to a specific pathogen, we analyze divergence in expression between species regardless of infections status.

Animals for this study were trapped in a dry deciduous wood (tree cover dominated by beech and oak) at Stensoffa field station, Revingehed, 20 km east of Lund, southern Sweden. Voles and mice were trapped during 5 days in August and September 2016 using live traps (Ugglan special, GrahnAB, Sweden). Traps were set in the evening, collected early in the morning, and immediately transported to the field station where selected animals were dissected without delay. To minimize variation in gene expression due to age, reproductive status, weather, etc., we focused on adult males (as indicated by the presence of a scrotum) and collected equal numbers of the two species on each day. To get a general measure of the immune gene expression of an animal, we analyzed spleen transcriptomes. The spleen is a lymphoid organ that harbors large numbers of immune cells, including monocytes and B and T lymphocytes, and plays an important role during the immune response to an infection (Lewis et al. 2019). Spleens were dissected within a couple of minutes of euthanization and stored in RNAlater RNA Stabilization Reagent (Qiagen) until RNA extraction. Samples were collected with permission from the Malmö/Lund board for animal experiment ethics (permission M47-14).

### RNA Sequencing

From each animal, about 30 mg of spleen tissue was homogenized with a TissueLyser II (Qiagen, GmbH, Hilden, Germany). Total RNA was extracted by RNeasy Mini Kit (Qiagen) and treated by RNase-Free DNase Set (Qiagen). RNA quality was assessed by measuring RNA Integrity Number (RIN) on a BioAnalyzer (Agilent, USA); in all cases RIN values were ≥8.8. Paired-end Illumina RNA sequencing was performed by SciLifeLab (Stockholm, Sweden) on a HiSeq2500 (Illumina) with HiSeq Control Software 2.2.58/RTA 1.18.64 and a 2 × 126 setup using ‘HiSeq SBS Kit v4’ chemistry.

### De Novo *Transcriptome Assemblies*

Reads were trimmed using Trimmomatic (Bolger, Lohse, and Usadel, 2014) with settings “2:30:10 SLIDINGWINDOW : 4:5 LEADING : 5 TRAILING : 5 MINLEN : 25.” All trimmed reads passed the quality test in FastQC0.11.5 (https://www.bioinformatics.babraham.ac.uk/projects/fastqc/). Trimmed reads were de novo assembled for bank vole and yellow-necked mouse using Trinity with default settings (Grabherr et al. 2011). CD-HIT version 4.6.8 (Fu et al. 2012) and Transdecoder (Haas et al. 2013) were used to cluster contigs with at least 95% similarity (-c 0,95) and to predict coding sequences, respectively. Potential assembly errors were reduced by mapping reads back to pre-filtered contigs using RSEM (Li and Dewey 2011) and removing contigs with TPM (transcripts per million mapped reads) values less than 1.

### Identification and Annotation of Orthologs

To identify orthologs between bank vole and yellow-necked mouse, a reciprocal best hit BLASTp was performed, using predicted amino acid sequences from bank vole and yellow-necked mouse filtered de novo transcriptome assemblies. Hits with an e-value < 1e-5 were selected to construct the putative one-to-one orthologs matrix. To annotate the putative orthologs, BLASTx (version 2.6.0) was used to match both bank vole and yellow-necked mouse transcripts to house mouse (*Mus musculus*) proteins (91,244 transcripts from 22,237 unique protein coding genes) downloaded from Ensembl (www.ensembl.org, release 87). Only orthologs that received the same annotation were retained.

### Quantification of Gene Expression

The reads from each species were mapped to their filtered de novo transcriptome assemblies using RSEM. Initial gene expression matrices were produced for the two species separately, including nonnormalized expected counts for each individual within species. Read counts for the annotated orthologs in the two species were extracted to make the full matrix and normalized using a trimmed mean of M-values (TMM) method in edgeR (Robinson et al. 2010). Fragments per kilobase per million mapped reads (FPKM; Trapnell *et al.*2010) were calculated and transformed to log_2_(FPKM). An empirical distribution of log_2_(FPKM) values for each individual was calculated by kernel density (Gaussian distribution) estimation in R version 3.5.2 (R Core Team, 2018), where after zFPKM values were computed according to the procedure described in Hart et al. (2013).

### Identification of Different Categories of Immune Genes

We compiled a list of immune genes likely expressed in spleen based on the following KEGG pathways (Kanehisa et al. 2017): FcγR-mediated phagocytosis; CLR signaling pathway; NOD-like receptor signaling pathway (excluding sensors and signaling genes exclusively involved in detection of DAMPs); RIG-I like receptor signaling pathway; TLR signaling pathway; NFkB signaling pathway; NK-cell-mediated cytotoxicity; BCR signaling pathway; TCR signaling pathway; Th1 and Th2 cell differentiation; Th17 cell differentiation; IL17 signaling pathway; TNF signaling pathway; and Chemokine signaling pathway. In addition, we included all cytokines and their receptors listed in appendix IV in Janeway’s Immunobiology (Murphy and Weaver 2017).

This yielded in total 732 genes. Of these, 676 could be divided into four functional categories (Table 1): genes encoding 1) PRR, which are involved in the initial detection of pathogens by recognizing microbe-associated molecular patterns (MAMP) like lipopolysaccharide and double-stranded RNA; 2) signal transduction proteins (adaptors, protein kinases, etc.), which mediate signaling downstream of PRRs and other receptors; 3) transcription factors (identified based on Animal TF database 3.0 [Hu et al. 2019]), which are activated by signaling proteins and induce expression of cytokines and other modulators of the immune response, and 4) cyto-/chemokines or cyto-/chemokine receptors (henceforth cytokines), which are involved in intercellular communication (autocrine, paracrine, and endocrine), thereby modulating immune responses. Most of the remaining 56 genes encoded different types of cell surface molecules (CD4, Fc receptor genes, etc.) that did not easily fit into any of the above categories, and were considered to have functions that were not similar enough to form a separate category; they were therefore excluded from further analyses. Our categories are similar to those used by Hagai et al. (2018), but we lumped all intracellular signal transduction proteins into one group to enhance statistical power, and because the analysis by Hagai *et al.* showed there was little difference in divergence in expression between subgroups of signaling proteins.

**Table 1.** Number of Immune Genes in Different Functional Categories

Function of Encoded Protein	Numberin KEGG Pathways	Number Annotatedin Both BV and YNMDe Novo TranscriptomeAssemblies	Number Used for Analyses ofSequence and ExpressionDivergence
Pattern recognition receptor (PRR)	44	22	22 (50%)
Signal transduction protein	364	193	193 (53%)
Transcription factor (TF)	53	27	27 (51%)
Cyto-/chemokines and their receptors	215	73	71 (33%)
Total	676	315	313 (46%)

Note.—BV, bank vole; YNM, yellow-necked mouse.

### Interspecific Divergence of Coding Sequences

Coding sequences of orthologs from the bank vole and yellow-necked mouse de novo transcriptome assemblies, as well as the longest transcript from *M. musculus* ortholog were aligned with PRANK version 170427 (Löytynoja 2014) using default settings except for specifying a codon-aware alignment, and parallelized using gnu-parallel (Tange 2011). All alignments were edited manually. In a few cases (*IL7R*, *MYD88*, *TBX21*, *TKFC*), the sequence from the bank vole transcriptome was complemented with sequence from the bank vole reference genome (Lundberg et al. 2020) to increase the overlap between bank vole and yellow-necked mouse. Following Warnefors and Kaessmann (2013), only genes with at least 150 bp of overlap between the aligned bank vole and yellow-necked sequences were used in analyses of sequence divergence. Only two of the immune genes (*CSF2RA* and *IL10RB*) did not fulfil this criterion, leaving *N* = 313 immune genes for analyses of coding sequence divergence. To obtain a set of control genes for comparison of coding sequence divergence, we selected *N* = 313 genes at random from the one-to-one orthologs not included in the list of immune genes (i.e., from 8599 − 315 = 8284 genes). These 313 control genes were aligned and edited in the same way as the immune genes. Coding sequence divergence of each pair of orthologs was estimated as the number of nonsynonymous substitutions per nonsynonymous site (*dN*) between bank vole and yellow-necked mouse (Warnefors and Kaessmann 2013). To test if any differences in *dN* between gene categories were a result of differences in mutation rate or selection, we also estimated the number of synonymous substitutions per synonymous site (*dS*), which is a crude measure of mutation rate (Pál et al. 2006), and *dN*/*dS* (where a relatively high value indicates relaxed purifying selection or positive selection). *dN* and *dS* were calculated with MEGA X for MacOS (Kumar et al. 2018; Stecher et al. 2020) using the Nei-Gojobori method with Jukes-Cantor correction. One gene (*ARPB1C*) had *dN* = 0 and *dS* = 0, and was assigned a *dN*/*dS* value of 0.

### Intraspecific Variation and Interspecific Divergence of Gene Expression

We used the standard deviation (SD) of zFPKM values as a measure of intraspecific variation (as the SD of a log-transformed variable is uncorrelated with the mean; Lewontin 1966).

Several different approaches have been used to estimate interspecific divergence in gene expression, including phenotypic correlations in gene expression between species (Brawand et al. 2011), principal component analysis (Uebbing et al. 2016), comparison of the relative variance in expression within and between species (Romero et al. 2012; Rohlfs and Nielsen 2015), comparison of genetic mean expression levels between species (Nourmohammad et al. 2017), and residuals from a regression of expression levels in different species (Chen et al. 2019). Some of these are useful for comparing divergence of sets of genes, for example in different tissues (Brawand et al. 2011; Uebbing et al. 2016), while others also give gene-specific estimates of divergence (Rohlfs and Nielsen 2015; Nourmohammad et al. 2017; Chen et al. 2019). Here, we employed the approach outlined by Chen et al. (2019), which yields gene-specific estimates of divergence. For this purpose, we used smatr3 (Warton et al. 2012) to extract residuals from a standardized major axis regression (where residuals are orthogonal to the regression line;Warton et al. 2006) of mean expression in bank voles against mean expression in yellow-necked mice, and used the squared residuals as a measure of divergence. SMA regressions were performed with all annotated orthologs, rather than just the immune genes, to obtain a more precise estimate of the regression between expression in bank voles and yellow-necked mice. Besides comparing expression divergence among the different categories of immune genes, we also compared each category to the set of 313 control genes (see above).

### Pleiotropy

Divergence in protein-coding sequences is often negatively correlated with pleiotropy (Zhang and Yang 2015). Similarly, Hagai et al. (2018) found that expression divergence was negatively correlated with pleiotropy (see also Papakostas et al.,2014). We therefore included pleiotropy as a potential covariate in analyses of differences in divergence in coding sequence and expression between gene categories. We used number of protein–protein interactions (PPI) as a proxy for pleiotropy (Papakostas et al. 2014; Zhang and Yang 2015). The number of PPI for *M. musculus* orthologs of the bank vole and yellow-necked mouse genes were retrieved from the STRING database (Szklarczyk et al. 2018), and were based on the sources “experiments” and “databases,” with confidence ≥0.4 (“intermediate confidence”).

### Statistical Analyses

For each variable, we compared the four immune gene categories against each other, using general linear models (GLM) and Tukey’s post hoc test. We also compared each of the immune gene categories to the set of control genes (see above), using GLM and Dunnett’s post hoc test. GLMs were performed with proc glm in SAS 9.4 (SAS Inc., Cary, USA). Squared residuals (from the SMA regressions) were log_10_transformed, while *dN* and *dN*/*dS* were arcsine squareroot transformed for the GLM analyses (*dS* was normally distributed). Continuous independent variables were Z transformed (mean = 0, SD = 1), so the effect of the factor immune gene category is tested at the mean value of each covariate (rather than 0).

## Results

### RNA-Sequencing

We performed RNA sequencing on spleen samples from 18 bank voles and 17 yellow-necked mice. Altogether, these 35 libraries generated ∼1.44 billion raw reads. De novo transcriptome assemblies contained 842,299 and 761,841 contigs for bank vole and yellow-necked mouse, respectively. A series of filtering steps resulted in the retention of 13,631 contigs and 13,744 contigs in bank vole and yellow-necked mouse, respectively (see Zhong et al. 2020 for more details regarding de novo transcriptome assemblies). The orthology between bank vole and yellow-necked mouse genes was determined by Reciprocal Best Hit (RBH) via BLASTp, which resulted in 10,931 one-to-one orthologs. Of these, 8,599 could be annotated (and received the same annotation) by BLASTx against a house mouse protein database.

Of the 676 categorized immune genes (see Material and Methods), 315 were included in our set of 8,599 annotated orthologs. Two of these had too short alignment length for calculating coding sequence divergence (<150 bp; see Materials and Methods); the remaining 313 represent the data set used in all analyses of divergence, etc., below (Table 1).

Plots of the frequency distributions of log_2_(FPKM) values for each individual showed that the data set was highly homogenous, with relatively little variation in mean and SD of log_2_(FPKM) between individuals (supplementary fig S1, Supplementary Material online). Nevertheless, there was a statistically significant difference in mean log_2_(FPKM) between bank voles and yellow-necked mice (*t*-test: *t* = 2.25, *df* = 33, *P = *0.03). We therefore applied the zFPKM transformation (Hart et al. 2013). A plot of the frequency distribution of log_2_(FPKM) expression values showed that there was no pronounced “shoulder” with lowly expressed genes (compare our Supplementary Fig 1 and Fig 2 in Hart et al. [2013]). We therefore retained all genes for further analyses, instead of excluding genes with zFPKM <−3 as recommended by Hart et al. (2013). In our data set, only 1 signaling and 3 cytokine genes had mean zFPKM <−3 in both bank voles and yellow-necked mice.

### Divergence of Coding Sequences

**Fig. 1.**
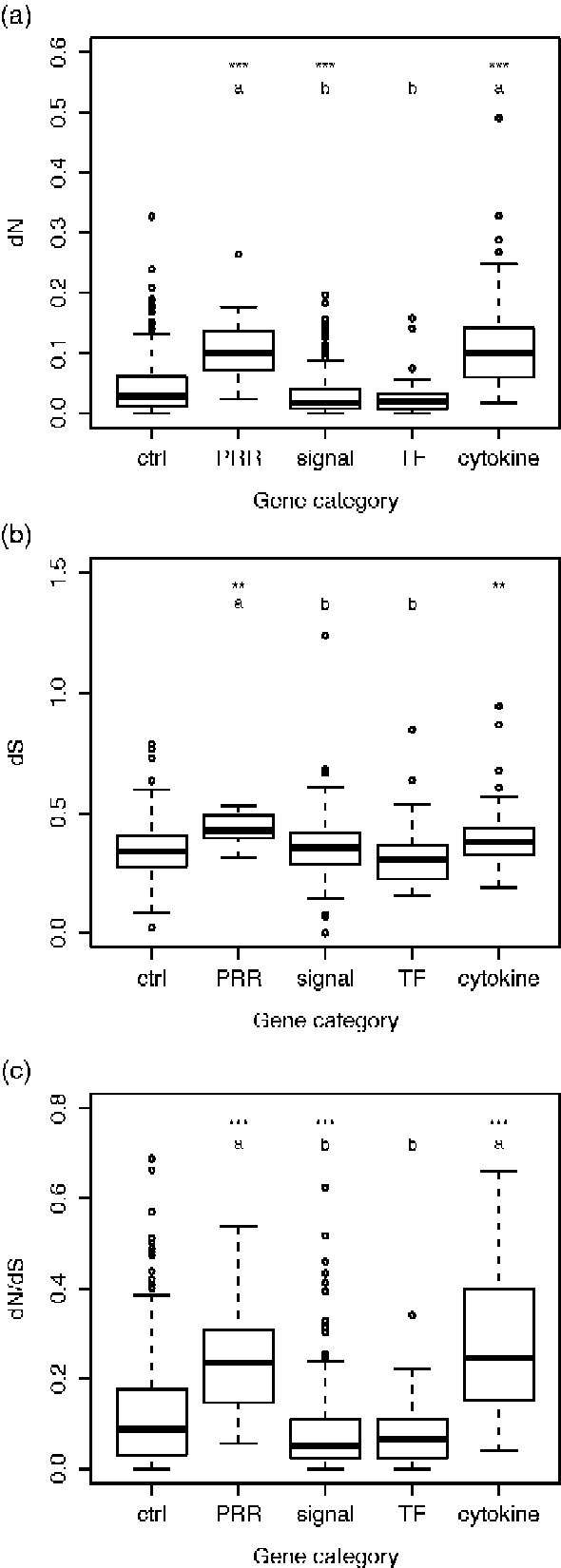
Divergence in coding sequence of different categories of immune genes and a set of randomly selected control genes. (*a*) The rate of nonsynonymous substitutions, *dN*. (*b*) The rate of synonymous substitutions, *dS*. (*c*) *dN*/*dS*. Letters indicate which categories of immune genes were significantly different from each other based on Tukey’s post hoc test; in panels (*a*) and (*c*), *P* in all cases <0.0001; in panel (*b*), *P < *0.05. Asterisks indicate which categories of immune genes were significantly different from the control genes based on Dunnett’s post hoc test: ***P <* *0.001, **P < 0.01.

The mean coding sequence divergence (estimated as the rate of nonsynonymous substitutions; *dN*) differed between categories of immune genes (*F* 3, 309 = 62.23. *P *<* *0.0001), with PRR and cytokine genes having higher *dN* than signaling and transcription factor genes (Tukey’s: *P *<* *0.0001; fig. 1*a*). A comparison of the mean *dN* of different categories of immune against the 313 randomly selected control genes showed that PRR and cytokine genes had higher *dN*, while signaling genes had lower *dN* than control genes (*F* 4, 621 = 45.08, *P *<* *0.0001, Dunnet’s: *P *≤* *0.01; fig. 1*a*).

Analyses of the rate of synonymous substitutions (*dS*, an estimate of the mutation rate) showed that PRR genes had higher *dS* than signaling and transcription factor genes (*F* 3, 309 = 4.80, *P *=* *0.0028, Tukey’s: *P < *0.05; fig 1*b*), and that PRR and cytokine genes had higher *dS* than control genes (*F* 4, 621 = 5.76, *P *=* *0.0001, Dunnett’s: *P *<* *0.01; fig 1*b*).

A comparison of the ratio of substitution rates (*dN*/*dS*, an estimate of the mode and strength of selection) among categories of immune genes revealed an identical pattern as for *dN*, with PRR and cytokine genes having higher *dN*/*dS* than signaling and transcription factor genes (*F* 3, 309 = 61.08, *P *<* *0.0001, Tukey’s: *P *<* *0.0001; fig 1*c*). PRR and cytokine genes also had higher *dN*/d*S* than control genes, while signaling genes had lower *dN*/*dS* than control genes (*F* 4, 621 = 40.48, *P* < 0.0001; Dunnett’s: *P *<* *0.001; fig 1*c*).

### Mean and Standard Deviation of Gene Expression

**Fig. 2.**
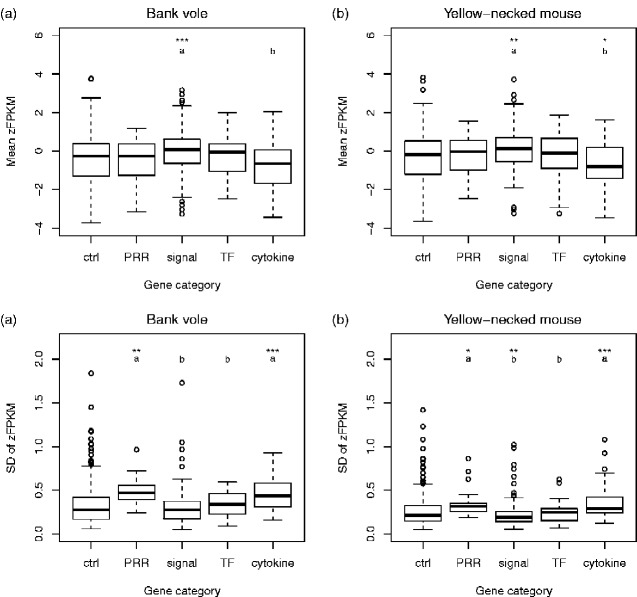
Mean and variation (SD) of expression levels (zFPKM) of different categories of immune genes and a set of randomly selected control genes in each species. (*a*) Mean expression in bank voles. (*b*) Mean expression in yellow-necked mouse. (*c*) SD of zFPKM in bank voles. (*d*) SD of zFPKM in yellow-necked mice. Letters indicate which categories of immune genes were significantly different from each other, based on Tukey’s post hoc test. Asterisks indicate which categories of immune genes were significantly different from the control genes based on Dunnett’s post hoc test: ***P <* *0.001, **P < 0.05, *P < 0.05.

The mean expression levels differed between immune gene categories, with signaling genes having significantly higher expression than cytokine genes in both bank voles (*F* 3, 309 = 8.95, *P *<* *0.0001, Tukey’s: *P* < 0.0001; fig 2*a*) and yellow-necked mice (*F* 3, 309 = 10.12, *P *<* *0.0001; fig 2*b*). Signaling genes also had higher expression than control genes in both bank voles (*F* 4, 621 = 6.90, *P *<* *0.0001, Dunnett’s: *P *=* *0.0002) and yellow-necked mice (*F* 4, 621 = 7.17, *P *<* *0.0001, Dunnett’s: *P *=* *0.0015). In yellow-necked mice, cytokine genes also had lower expression than control genes (*P = *0.017).

The intraspecific variation in gene expression (measured as the standard deviation of zFPKM) differed between immune gene categories, with PRR and cytokine genes having higher level of variation than transcription factor and signaling genes in both bank voles (*F* 3, 309 = 18.62, *P *<* *0.0001, Tukey’s: *P < *0.05; fig 2*c*) and yellow-necked mice (*F* 3, 309 = 21.32, *P *<* *0.0001, Tukey’s: *P < *0.05; fig 2*d*). PRR and cytokine genes also had higher level of variation than control genes in both bank voles (*F* 4, 621 = 8.51, *P *<* *0.0001, Dunnett’s: *P ≤ *0.0077) and yellow-necked mice (*F* 4, 621 = 10.90, *P < *0.0001, Dunnett’s: *P ≤ *0.043). In yellow-necked mice, signaling genes also had lower SD than control genes (*P = *0.0054).

### Divergence of Gene Expression

**Fig. 3.**
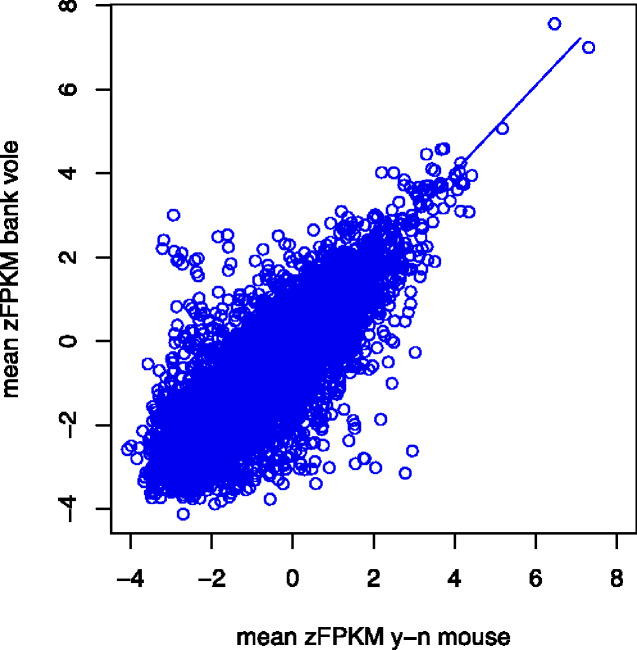
Standardized major axis (SMA) regression of mean expression (zFPKM) in bank vole against mean expression in yellow-necked mice based on 8599 orthologs.

Mean gene expression across all 8,599 orthologs in the yellow-necked mouse and bank vole de novo transcriptome assemblies were strongly correlated (*r*_s_=0.797, *P < *0.001). We used the squared residual deviation in a SMA regression of expression in bank voles against expression in yellow-necked mice as a measure of divergence (see Materials and Methods for details). The intercept and slope of a SMA regression of bank vole against yellow-necked mouse were −0.054 ± 0.018 and 1.025 ± 0.013 (±95% CI), respectively (fig. 3).

**Fig. 4.**
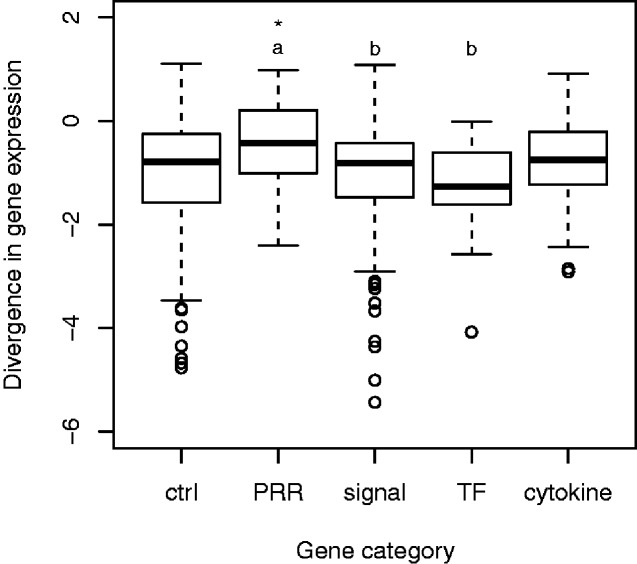
Divergence in gene expression between bank vole and yellow-necked mice for different categories of immune genes, where divergence is measured as the squared residual deviation (log_10_) in a SMA regression of mean expression in bank voles against mean expression in yellow-necked mice. Letters indicate which categories of immune genes were significantly different from each other based on Tukey’s post hoc test (PRR vs signal: *P < *0.01; PRR vs TF: *P *<* *0.05). The asterisk indicate which categories of immune genes is significantly different from the control genes based on Dunnett’s post hoc test: * *P < *0.05.

The extent of interspecific divergence in gene expression differed between immune gene categories (*F* 3, 309 = 4.60, *P *<* *0.0037), with PRR genes having higher divergence than signaling and transcription factor genes (Tukey’s: *P *≤* *0.021; fig. 4). PRR genes also had higher divergence than control genes (*F* 4, 621 = 3.16, *P = *0.014, Dunnett’s: *P = *0.034).

### Divergence of Coding Sequence and Expression in Relation to Other Factors

**Fig. 5.**
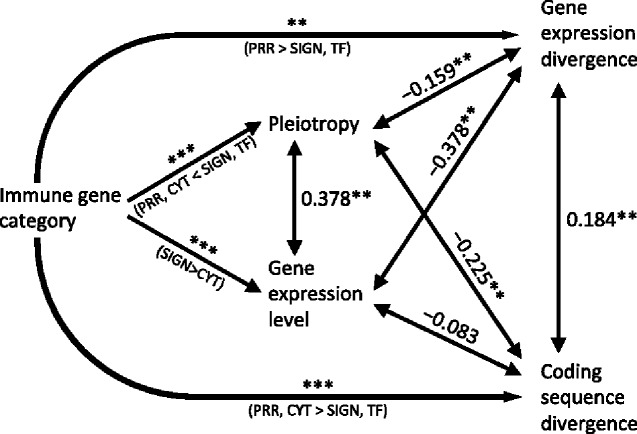
Path model showing the effects of the factor immune gene category on the four continuous variables pleiotropy, gene expression level (mean of mean expression in bank voles and yellow-necked mice), gene expression divergence, and coding sequence divergence, as well as correlations among the four continuous variables. Asterisks above arrows from the factor immune gene category indicate the results of GLMs with the continuous variable against immune gene category; text below arrows indicate results of Tukey’s post hoc tests (PRR, pattern recognition receptor genes; SIGN, signalling genes; TF,transcription factor genes; CYT,genes encoding cytokines, chemokines, and their receptors). Values at bidirectional arrows indicate Spearman rank correlation coefficients. ***P < 0.001; **P < 0.01.

To illustrate how sequence and expression divergence are related to other factors (including each other), we constructed a path model involving the following variables: immune gene category, gene expression level (mean of mean expression in voles and mice), pleiotropy, gene expression divergence, and coding sequence divergence (fig. 5). Besides the effects of immune gene category on sequence divergence and expression divergence already reported above, this factor also had significant effects on pleiotropy (GLM: *F* = 23.68, *df* = 3, 309, *P < *0.0001; fig 5) and expression level (GLM: *F *= 10.64, *df* = 3, 309, *P < *0.0001; fig 5). In addition, five out of six of the pairwise correlations between continuous variables were statistically significant (fig. 5).

To test if the difference in sequence divergence between immune gene categories (fig 1*a*) occurred independently of other differences between gene categories, we performed a GLM with sequence divergence against the factor immune gene category, the covariates expression level, expression divergence, and pleiotropy, as well as the two-way interactions between gene category and the covariates. This showed that the effect of gene category (i.e., the difference in *dN* between genes encoding PRR and cytokines on one hand and those encoding signaling proteins and transcription factors on the other) remained highly significant (*F* = 42.9, *df *= 3, 304, *P < *0.0001) even when controlling for a negative correlation between sequence divergence and pleiotropy (*F* = 5.04, *df* = 1, 304, *P = *0.026), and an interaction between gene category and expression divergence (*F* = 3.41, *df *= 3, 304, *P = *0.018; see supplementary table S1, Supplementary Material online for full model details). Also the differences in *dN* between PRR and cytokine genes on one hand and control genes on the other (fig 1*a*) remained when controlling for potentially confounding factors (supplementary table S2, Supplementary Material online). Similar analyses for *dS* and *dN*/*dS* showed that differences between gene categories were independent of covariates also for these variables (supplementary tables S3–S6, Supplementary Material online).

To test if the difference in gene expression divergence between immune gene categories (fig. 4) occurred independently of other differences between gene categories, we performed a GLM with expression divergence against gene category, the continuous independent variables pleiotropy, expression level, and sequence divergence, and the two-way interactions between gene category and the continuous independent variables. This showed that there was a significant interaction between gene category and sequence divergence (*F* 3, 305 = 3.09, *P = *0.027). When controlling for gene category × sequence divergence, the difference in expression divergence among gene categories (fig. 4) was no longer significant (*F* 3, 305 = 2.50, *P = *0.06; see supplementary table S7, Supplementary Material online for full model details). The difference in expression divergence between PRR and control genes (fig. 4) did however remain significant even when controlling for a negative correlation between expression divergence and mean expression (supplementary table S8, Supplementary Material online).

### Correlation between Expression Divergence and Coding Sequence Divergence

**Fig. 6.**
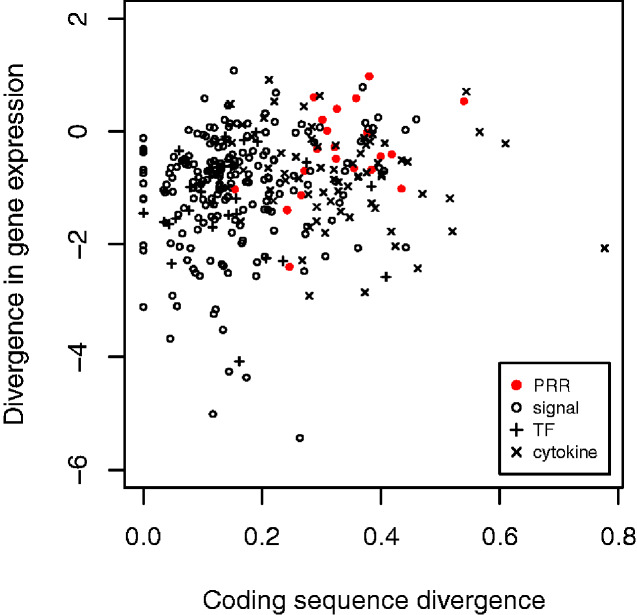
Expression divergence (log_10_ transformed) against coding sequence divergence (*dN*, arcsin squareroot transformed) for different categories of immune genes.

The GLMs of both coding sequence and expression divergence showed statistically significant interactions between gene category and expression divergence or sequence divergence (see above), indicating that the strength of the association between expression divergence and sequence divergence varied among immune gene categories. To examine this in more detail, we calculated Spearman rank correlations between expression divergence and sequence divergence for each immune gene category. There were significant positive correlations between expression divergence and sequence divergence for PRR (*r*_s_=0.477, *P = *0.025) and signaling genes (*r*_s_=0.159, *P = *0.027), but not for cytokine and transcription factor genes (*r*_s_=−0.187, *P = *0.12, and *r*_s_=0.14, *P = *0.49, respectively; fig. 6).

## Discussion

Here, we divided genes in immune signaling pathways into four broad functional categories and tested if they differ in degree of coding sequence and expression divergence between two rodent species.

Genes encoding PRR and cytokines had considerably higher coding sequence divergence (*dN*) than genes encoding signal transduction proteins and transcription factors. The sequence divergence of PRR and cytokine genes was also higher than that of a set of nonimmune control genes. The differences between categories remained even after controlling for some potentially confounding factors (mean expression level, pleiotropy, and expression divergence). We acknowledge that there is likely considerable noise in the estimates of the covariates. For example, gene expression was measured in only one organ (spleen), and pleiotropy was estimated as PPI in a different species (*Mus musculus*) and even in that species knowledge is incomplete. In addition, there might be other factors that could explain the difference in sequence divergence between gene categories (Zhang and Yang 2015). Nevertheless, given the strong independent effect of gene category, it seems likely there is an effect of functional category per se on coding sequence divergence. Genes encoding PRR and cytokines not only had higher *dN* but also higher *dS* than other gene categories. An association between *dN* and *dS* is a common finding in mammals and could have a number of different causes, for example correlated selection on adjacent synonymous and nonsynonymous sites (Smith and Hurst 1999, Stoletzki and Eyre-Walker 2011).

Analyses of *dN*/*dS* showed that differences in coding sequence divergence between gene categories was an effect of differences in selection between PRR and cytokines on one hand and signal transduction and transcription factor genes on the other, with PRR and cytokine genes having experienced relaxed purifying or enhanced positive selection relative to signal transduction and transcription factor genes. These two explanations could be distinguished by tests based on codon models of sequence evolution (Kosiol and Anisimova 2012), but that would require a data set with more than two species. Previous analyses of other mammals have shown that genes encoding both PRR and cytokines often have signatures of positive selection (Kosiol et al. 2008; Wlasiuk and Nachman 2010; Neves et al. 2014), suggesting that the high sequence divergence of such genes between the bank vole and yellow-necked mouse is a result of positive selection rather than relaxed constraint. Analyses of bank voles and humans have shown that PRR and cytokine genes also have relatively high levels of intraspecific diversity (Casals et al. 2011; Lundberg et al. 2020), and that the elevated diversity is at least partly a result of balancing selection (Lundberg et al. 2020).

Positive selection on receptors for recognition of pathogens has been attributed to that these proteins interact directly with pathogens and therefore are more likely to be involved in host–pathogen coevolution than other immune genes (Sackton et al. 2007). It is indeed easy to envision that selection on pathogens for evading recognition and subsequent positive selection on host recognition receptors to “chase” evolving pathogens can lead to a high rate of evolution of such receptors. In contrast, it is not immediately clear why there should be positive selection on genes encoding cytokines. One possibility is that these proteins are targets of pathogen immune evasion, and thus have a high rate of evolution due to selection for avoiding interference by pathogen proteins (Finlay and McFadden 2006). For example, some viruses produce decoy cytokine receptors that neutralize cytokines (Jensen 2017). It should be noted, though, that immune evasion also often targets intracellular signaling proteins (Finlay and McFadden 2006; Hoffmann et al. 2015), but genes in this category on average have much lower sequence divergence than cytokine genes (although there are a number of outliers in the signaling category [fig. 1*a–c* ]; these could potentially be a result of selection imposed by immune evasion).

The generally high divergence of coding sequences of PRR genes likely plays an important role in interspecific variation in immune function, by influencing the ability of a host species to recognize and respond to a given pathogen. Indeed, there are several cases where substitutions in the coding sequence of PRR genes have been pinpointed as key determinants of interspecific variation in resistance to particular pathogens (Werling et al. 2009; Palesch et al. 2018; Adrian et al. 2019), although there is also at least one example of that substitutions in a signaling gene play an important role (Xie et al. 2018). The contribution of the high divergence in coding sequences of genes encoding cyto-/chemokines or cyto-/chemokine receptors to interspecific variation in immune function is more questionable. If a change in the coding sequence of a cytokine receptor (driven by pathogen immune evasion) is followed by a compensatory change in the coding sequence of the corresponding cytokine that restores cytokine—receptor affinity, so that cytokines and their receptors are coadapted, high sequence divergence could have limited effects on immune function.

Overall, differences in expression divergence between categories of immune genes were less pronounced than in the in vitro study of divergence in response to stimulation (Hagai et al. 2018), presumably as a consequence of that our data set is more noisy because individuals harbored different infections at the time of sampling. Nevertheless, as in Hagai et al. (2018), PRR genes had higher expression divergence than some of the other immune gene categories (here signaling and transcription factors). In apparent contrast to Hagai et al. (2018), cytokines did not differ in expression divergence from signaling and transcription factors. However, this is probably at least partly a result of cytokine genes having highly variable expression within species in our data set, which obscures differences in mean expression level between species. When controlling for potentially confounding factors (of which the gene category × sequence divergence interaction was statistically significant), the difference in expression divergence between PRR genes and other immune gene categories was marginally nonsignificant. Thus, there are at least not strong differences in expression divergence between immune gene categories independently of other factors.

The analyses of coding sequence divergence and expression divergence revealed a correlation between these variables, and that the strength of the correlation varied between gene categories, being particularly strong for PRR. A previous study found that the strength of this correlation varied among organs, being strongest in brain and weakest in liver and testis (Warnefors and Kaessmann 2013). What is the cause of the correlation between sequence and expression divergence, and why is it strongest for PRR genes? At least two different scenarios have been proposed to explain the occurrence of a correlation between sequence and expression divergence in general (Warnefors and Kaessmann 2013). First, both sequence and expression divergence could be governed by some other factor. For example, previous studies have shown that both sequence and expression divergence tend to be negatively correlated with expression level (Warnefors and Kaessmann 2013). However, controlling for expression level did not affect the correlation between sequence divergence and expression divergence in either (Warnefors and Kaessmann 2013) or the present study. Second, sequence divergence and expression divergence might be associated because selection acts in similar ways (purifying/positive) on the coding sequence and *cis*-regulatory elements of a gene; this explanation is supported by analyses of both *Drosophila* and vertebrates (Lemos et al. 2005; Warnefors and Kaessmann 2013). Still, it is not obvious why it should result in that the correlation is particularly strong for PRR genes. We therefore propose an additional explanation which applies specifically to PRR, namely that divergence in PRR coding sequence causes divergence in PRR expression. Signaling by a PRR does not only affect expression of cytokines and other modulators of the immune response, but also often leads to upregulation of expression of the PRR itself (e.g.,*TLR2* and other PRR genes in response to *Borrelia* [Petzke et al. 2016]; see also [Hagai et al. 2018]). Thus, if high coding sequence divergence leads to that PRR binding affinity to a given pathogen ligand (MAMP) differs between species, this could lead to high divergence in expression of the PRR too. While the ‘similar selection’ scenario provides a general explanation for the correlation between sequence and expression divergence, an additional causal effect of PRR sequence divergence on PRR expression divergence could explain why the correlation is particularly strong for this category.

To sum up, we found that PRR and cytokine genes stand out as having particularly high coding sequence divergence. Of these, at least the high divergence of PRR genes is likely to play a major role in differences in immune function between species. PRR genes also had relatively high expression divergence, but we have interpreted this as being an indirect phenotypic effect of other factors, in particular coding sequence divergence, rather than a direct effect of divergent selection on expression of PRR genes.

Besides divergence in coding sequence and expression of orthologs, differences in immune function between species could be caused by interspecific variation in gene content as a result of gene duplication and subsequent neo-/subfunctionalization of paralogs (Kaessmann 2010; Sackton 2019). Analyses of *Drosophila* showed that some categories of immune genes (effector and recognition genes) had higher copy number variation across species than others (Sackton et al. 2007). It is well-known that some types of vertebrate-specific immune genes, like MHC and immunoglobulin genes, have high rates of duplication and considerable copy number variation between species, and it seems likely there are differences in duplication rate also between the immune gene categories considered in the present study. However, such an analysis would require high-quality reference genomes and can therefore not yet be performed with our study species. Gene duplication can be expected to affect both coding sequence divergence and expression divergence (Zhang et al. 2003; Pegueroles et al. 2013; Guschanski et al. 2017); it would therefore be of great interest to expand the present analyses by investigating the interplay between gene duplication, coding sequence divergence and expression divergence in the evolution of immune function.

## Supplementary Material

Divergence2_Supplementary Material are available at *Genome Biology and Evolution* online (http://www.gbe.oxfordjournals.org/).

## Author Contributions

X.Z. and L.R. performed field work. X.Z. performed lab work. X.Z. and M.L. performed bioinformatic analyses. L.R. performed statistical analyses. L.R. and X.Z. wrote the article with input from M.L.

## Data deposition

The sequence data have been deposited in the Sequence Read Archive (SRA) database at the National Center for Biotechnology Information (NCBI) under the BioProject PRJNA556160. Alignments of immune genes and the processed data set have been provided at Dryad (https://doi.org/10.5061/dryad.ncjsxkss3).

## Supplementary Material

evab023_Supplementary_Data
